# Intraspecific variation in the petal epidermal cell morphology of *Vicia faba* L. (Fabaceae)

**DOI:** 10.1016/j.flora.2018.06.005

**Published:** 2018-07

**Authors:** Emily J. Bailes, Beverley J. Glover

**Affiliations:** a School of Biological Sciences, Royal Holloway University of London, Egham, Surrey, TW20 0EX, United Kingdom; b Department of Plant Sciences, University of Cambridge, Downing Street, Cambridge, CB2 3EA, United Kingdom; c National Institute of Agricultural Botany, Huntington Road, Cambridge, CB3 0LE, United Kingdom

**Keywords:** Broad bean, Cell shape, Faba bean, Field bean, Papillose cell, Petal epidermis

## Abstract

At a microscopic scale, the shape and fine cell relief of the petal epidermal cells of a flower play a key role in its interaction with pollinators. In particular, conical shaped petal epidermal cells have been shown to have an important function in providing grip on the surface of bee-pollinated flowers and can influence bee visitation rates. Previous studies have explored interspecific variation in this trait within genera and families, but naturally-occurring intraspecific variation has not yet been comprehensively studied. Here, we investigate petal epidermal cell morphology in 32 genotypes of the crop *Vicia faba*, which has a yield highly dependent on pollinators. We hypothesise that conical cells may have been lost in some genotypes as a consequence of selective sweeps or genetic drift during breeding programmes. We find that 13% of our lines have a distribution of conical petal epidermal cells that deviates from that normally seen in *V. faba* flowers. These abnormal phenotypes were specific to the ad/abaxial side of petals, suggesting that these changes are the result of altered gene expression patterns rather than loss of gene function.

## Highlights

• Three different distributions of conical cells across *Vicia faba* petals were identified.
• The most common phenotype was present in 28/32 genotypes.
• Total loss or gain of conical cells was never observed.

## Graphical abstract

**Figure:**
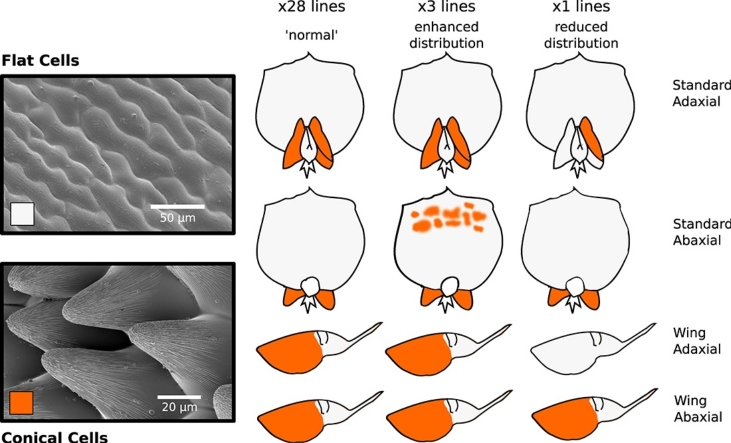

## 1 Introduction

The majority of flowers rely on animals, particularly insects, for their pollination. This has led to a magnificent array of flower colours, scents and shapes which maximise the reproductive fitness of species with diverse pollinators (Faegri and van der Pijl, 1979). One trait that is less well known with respect to its influence on pollinator visitation rates is that of the fine scale surface structure of the petal. The epidermis of plants is highly variable in morphology, with different cell shape and cell surface textures resulting from cuticle folding and ornamentation with other compounds such as epicuticular waxes (Koch et al., 2008). These different cell structures influence the interaction of plants with pathogens, pests and mutualists by altering the grip and accessibility of the surface, as well as its optical properties (Gorton and Vogelmann, 1996; Comba et al., 2000; Whitney et al., 2009; Alcorn et al., 2012).

One particular cell morphology that influences the interaction of a flower with its pollinators is the presence of conical petal epidermal cells. These cone-shaped cells are found on the petals of 75–80% of angiosperms analysed (Kay et al., 1981; Christensen and Hansen, 1998). Bees have been shown to have a preference for flowers with conical epidermal cells (Glover and Martin, 1998), especially when flowers are more difficult to manipulate, because they improve grip on the surface (Whitney et al., 2009; Alcorn et al., 2012). This increased grip will reduce the energy expenditure required to feed from a flower. Conical cells have been suggested to increase the temperature of flowers (Comba et al., 2000), although there is debate about the extent and significance of this effect (Whitney et al., 2011a). Therefore, conical cells may further reduce the energy expenditure of bees by reducing their need to use muscle shivering to maintain their body temperature (Heinrich and Esch, 1994). From an advertising perspective, conical cells are also known to benefit a flower by enhancing its colour by focusing light onto the floral pigments (Noda et al., 1994; Gorton and Vogelmann, 1996). It has also been suggested that conical cells, which reduce the wettability of the flower surface, act as a self-cleaning mechanism to keep flowers free of dust and other particles which may make their surface less attractive to pollinators (Whitney et al., 2011b).

Bilaterally symmetrical flowers such as those found in most legumes are particularly interesting when investigating the function of petal epidermal cell morphology because of the specific way pollinators interact with these petals. Fabaceae flowers are generally organised into three petal types: the dorsal standard, lateral wing and ventral keel petals. The wing and keel petals are joined at their base by petal folds. During a legitimate visit, a bee alights on the wing petals and pushes downwards on the wing petals to allow access to the nectar at the base of the flower and pollen contained on the anthers and within the keel petals (Stoddard, 1991). The standard predominantly acts as an advertisement to pollinators.

**Fig. 1:**
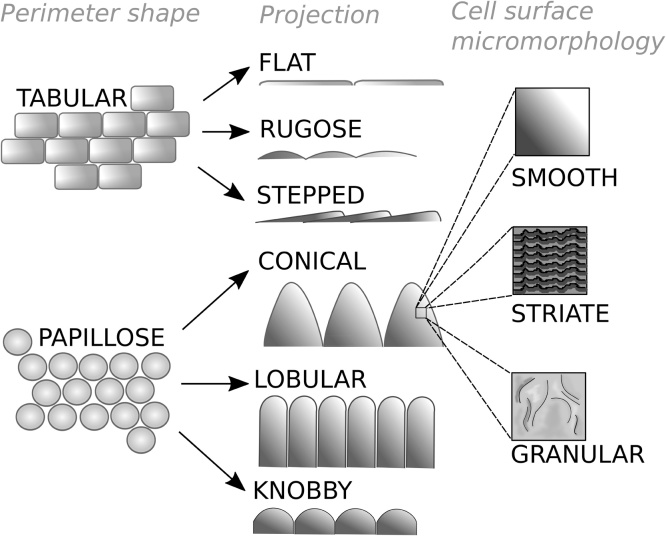
The classification of the protruding parts of epidermal cell morphology. Epidermal cells can be classified based on three levels, the shape of the cell perimeter (Perimeter Shape), the amount of projection from the cell surface (Projection), and the micromorphology of the cell surface (Cell surface micromorphology).

A large-scale analysis of flower epidermal cell morphology in the Fabaceae identified six main categories of cell types (Fig. 1) based on both their primary (cell shape) and secondary structure (cell wall fine relief); tabular rugose granular, tabular rugose striate, tabular flat striate, papillose conical striate, papillose knobby rugose, and papillose lobular striate (Ojeda et al., 2009). This study suggested that certain cell types are associated with the standard, wings and keel petals in Fabaceae. For example, papillose conical striate cells (conical cells) are generally a feature of the standard and wing but not keel petals in the most derived subfamily, the Papilionoideae (Ojeda et al., 2009). Given that the keel petal plays more of a functional role in containing the pollen of the flower rather than directly interacting with or attracting pollinators, this distribution of cell morphology within the flowers of the Papilionoideae is therefore not surprising.

Previous investigations into the distribution of petal epidermal cell morphology have largely focused on differences between broad taxonomic groups (Kay et al., 1981; Christensen and Hansen, 1998; Papiorek et al., 2014) or within specific families (Baagøe, 1977, 1980; Ojeda et al., 2009). A handful of studies have also examined differences in petal epidermal morphology within genera, particularly in genera with more than one functional group of pollinators (Di Stilio et al., 2009; Çildir et al., 2012; Ojeda et al., 2012, 2016). From these previous studies we know that substantial variation can occur in the petal epidermal cell types present between flowers of different species, even within a genus. This is particularly true when species evolve associations with non-insect pollinators. For example, in all five cases of shifts from insect to bird pollination in Macaronesia (in *Lotus* spp. L. (Fabaceae), *Anagyris latifolia* Brouss. ex Willd. (Fabaceae), *Navaea phoenicea* Webb & Berthel. (Malvaceae), *Isoplexis* spp. (Lindl.) Loudon (Plantaginaceae), and *Canarina canariensis* (L.) Vatke (Campanulaceae)) the transition is associated with the loss of conical cells (Ojeda et al., 2016). More generally, bird pollination is associated with quantitatively flatter petal epidermal cells than in bee pollinated flowers, which may help to deter nectar robbing bees (Papiorek et al., 2014). Similarly, in *Thalictrum* Tourn. ex L. transitions from insect to wind pollination are also associated with the loss of conical cells (Di Stilio et al., 2009). However, despite these investigations into the distribution of petal epidermal cell types across flowering plant families, there is little discussion of intraspecific variation. Those studies that have examined multiple individuals from a single species have generally found no significant variation between individuals (Ojeda et al., 2009; Çildir et al., 2012), with the exception of two subspecies of *Echium wildpretii* H.Pearson ex Hook.f. with different functional groups of pollinators (Ojeda et al., 2016). However, these studies had limited sample sizes (2–6 individuals per species), and no explicit intention to sample across the genetic diversity of a species, and may therefore underrepresent the diversity of epidermal phenotypes found within a species.

Crop plants present an ideal opportunity to explore the presence of intraspecific variation in petal epidermal morphology, because many independent genotypes are retained in stock centres for commercial breeding. Crops such as the field bean *Vicia faba* L. are dependent on pollinators for maximum yield (Klein et al., 2007; Cunningham and Le Feuvre, 2013; Garratt et al., 2014). However, floral traits are rarely selected for in breeding programmes and therefore may have become suboptimal for maximizing pollination through genetic drift (Kobayashi et al., 2010; Bailes et al., 2015). Previously it has been reported that the major epidermal cell type present on *V. faba* standard and keel petals are tabular cells, whereas the wing petals mainly display conical cells (Ojeda et al., 2009). We were interested in determining whether this phenotype was consistent between different genotypes of *V. faba* or whether intraspecific variation, potentially providing an opportunity for selective breeding to improve pollinator attraction, was present. We examined the petal epidermal cell morphology of the apical (pollinator-contacting) portion of cells for 32 genetically distinct lines of *V. faba* and asked (i) which cell types are present within *V. faba* flowers? and (ii) is there variation in the distribution of conical petal epidermal cells between genetically distinct lines of *V. faba*?

## 2 Methods

### 2.1 Plant material

To determine the level of variation in epidermal cell morphology within *V. faba* we randomly selected 32 lines from the seed collections at the National Institute of Agricultural Botany (Sources in Table S1). These lines had been self-pollinated for at least 5 generations and therefore should be homozygous at the majority of loci. The majority of the lines were white with black wing-petal spots, as is typical for field bean flowers. However, lines NV175, NV643, NV644, NV676 and NV868 lack wing-petal spots, and are pure white. Line NV706 had a crimson flower with dark wing-petal spots. Vouchers for specimens of a plant from each line used in the study were deposited in the University of Cambridge herbarium (Cambridge, UK), with the voucher numbers CGE33556 – CGE33587 (Table S1).

### 2.2 Sampling strategy

For each line, one flower was analysed to represent that genotype, as petal epidermal cell type has never been shown to be influenced by environment. The pollinator-interacting wing and standard petals were imaged for all 32 lines, focusing on the distribution of conical cells in these petals. For a subset of five of these lines a more in depth analysis of the cell types present was undertaken, including of the keel petals.

### 2.3 Imaging

Dental wax (Zhermack Elite HD + Dental wax, Light body) casts of fresh fully open flowers were made for both the adaxial and abaxial surface of all petals of interest by pressing each petal into freshly mixed wax then peeling the petal away once the wax was set. This method preserves the native structure of the petal surface and reduces the risk of introducing artefacts compared to tissue preparation processes that use dehydration. From these, epoxy-resin replicas were produced using 2 Ton Epoxy (DevCon), and sputter coated with gold or platinum using a Quorum K756X sputter coater. Surface replicas were examined using a FEI Philips XL30 Scanning Electron Microscope. Petals were surveyed for the absence or presence of conical cells at the apical part of the cell and the distribution of the cell types noted.

### 2.4 Epidermal cell morphology classification

Epidermal cell types were classified into discrete categories following Christen and Hansen (1998) and Ojeda et al. (2009). These categories encompassed aspects of the overall cell morphology and relief at the apical part of the cell where pollinators will make contact with the petal. The categories are defined in this study as follows: ‘tabular’ describes cells that were jigsaw-shaped or were roughly rectangular in their perimeter. ‘Papillose’ cells were roughly circular or oval shaped in their perimeter. Within the ‘papillose’ cells there were three subtypes ‘conical’, ‘lobular’ or ‘knobby’ which were identified by Ojeda et al. (2009) in legume flowers. Here, ‘conical’ describes a cone shaped protrusion from the surface, ‘lobular’ a roughly cylindrical shaped protrusion, and ‘knobby’ where protrusions are only very short, with very little space between them so that a pavement pattern is formed (Fig. 1). Within ‘tabular’ cells, the cell morphology could be ‘flat’, ‘rugose’ or ‘stepped’. Unlike ‘flat’ cells ‘rugose’ cells were raised or ridged, so that a hypothetical transverse-section would be semi-circular in shape. ‘Stepped’ are newly defined in this study as flat cells where rows overlapped at one edge to form a step-like structure. Finally, a cell could have fine sculpturing that was ‘smooth’ where there were no micro-protrusions from the surface, ‘striate’ where the epidermis formed ridges or ‘granular’ where the epidermis formed non-linear protrusions. Where cells were classed as ‘non-conical’, they were not cone shaped, including domed-shaped (lobular of knobby) non-flat cells.

## 3 Results

### 3.1 An in-depth investigation into the distribution of epidermal cell types within a flower of *V. faba*

**Fig. 2:**
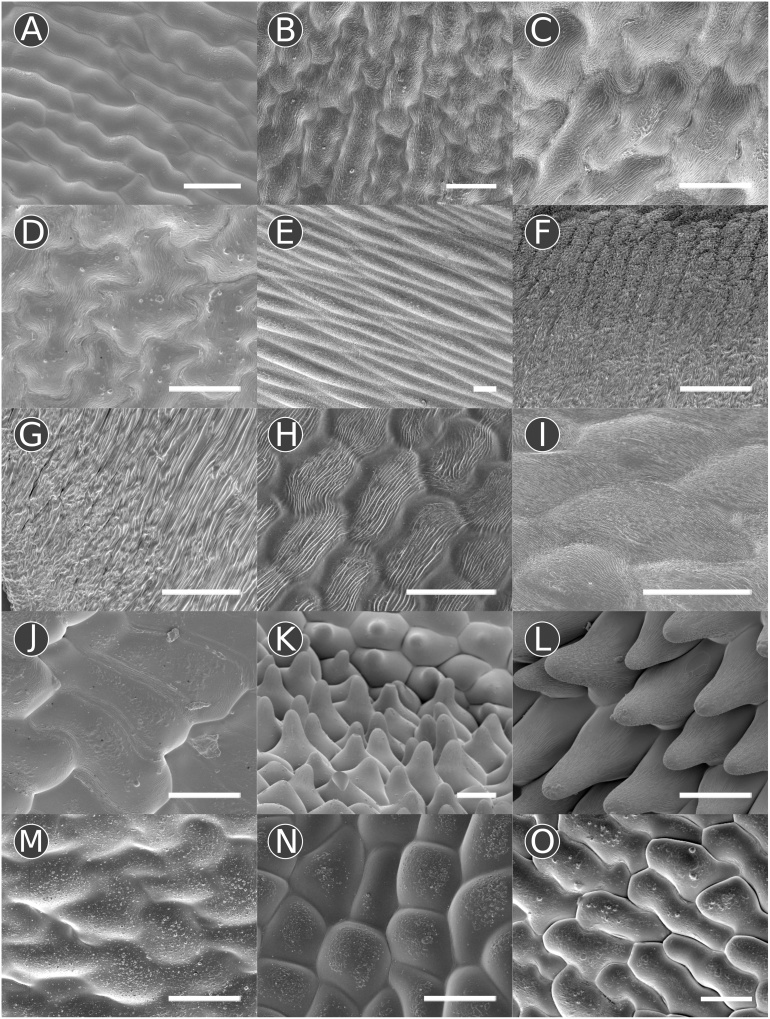
Examples of epidermal cell types found in *V. faba*. (A–E) tabular rugose striate cells on the (A) standard (adaxial) of NV644, (B) standard (abaxial) of NV648, (C) keel (adaxial) of NV640, (D) keel (abaxial) of NV648, and (E) standard of NV644 (abaxial) – these elongated cells are characteristic of the base of the standard petal. (F & G) tabular rugose granular cells on the (F) keel (adaxial) of NV648 and keel (abaxial) of NV644. (H & I) tabular flat striate cells on the (H) keel (adaxial) of NV706 and (I) keel (abaxial) of NV648. (J) Tabular stepped striate cells from the wing (abaxial) of NV644, these cells are characteristic of the adaxial surface of wing petals. (K & L) papillose conical striate cells on the (K) wing (adaxial) of NV643 and (L) wing (abaxial) of NV643. (M–O) tabular rugose smooth cells on the (M) standard (adaxial) of NV644, (N) keel (abaxial) of NV706 and (O) wing (abaxial) of NV706. Scale bars are 50 μm long.

**Fig. 3:**
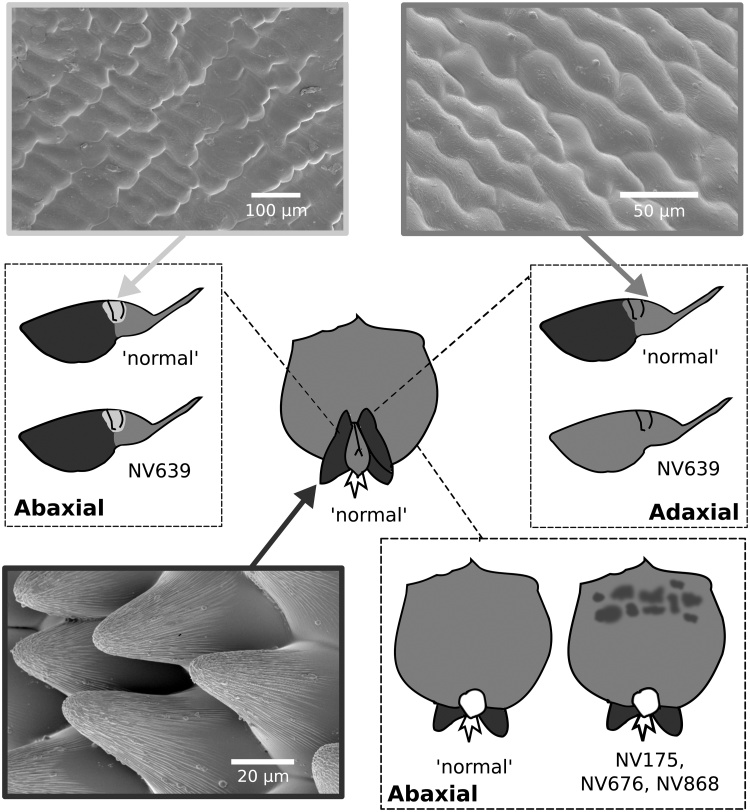
Variation in the distribution of the major cell types in *V. faba*. Examples of ‘Stepped cells’ and ‘Conical cells’ are of the abaxial surface of a wing petal in NV644, ‘Tabular cells’ are of the adaxial surface of the standard petal in NV640. Schematics of the different distributions of conical cells found in the flowers of *V. faba* in this study are given with the most prevalent distribution indicated as ‘normal’ and lines with a different distribution listed. All lines had flat cells across the adaxial surface of the standard petal. Dark grey indicates the presence of conical cells, light grey indicates stepped cells, and mid-grey indicates other tabular (non-conical) cell types.

An initial survey of the distribution of conical cells across the adaxial and abaxial surface of all three petal types in lines NV640, NV643, NV644, NV648 and NV706 revealed no differences in the distribution of cell types present between these lines. The three different petal morphologies produced by *V. faba* – standard, wing and keel petals – had specific categories of cell types associated with them so that they could be discriminated on the basis of their epidermal cell morphology (Table 1; Fig. 2; see methods for descriptions of terminology). In particular, the keel petals could be distinguished from other petals by a region of tabular rugose granular cells, which were located in a narrow band at the dorsal edge on both sides of the petal. Below this region, tabular flat striate cells were present, a cell type unique to the keel petals. The remainder of the epidermal cells of the keel petals, on both sides, were predominantly tabular rugose striate or tabular rugose smooth. The wing petals could be distinguished from other petal types by the presence of papillose conical striate cells, which constituted the majority of the cells present on both sides and were distributed continuously from the petal tip inwards towards the base, phasing out into tabular rugose smooth cells just before the petal folds at the base of the wing petal (Fig. 3). In general, papillose conical striate cells were distributed over a larger proportion of the petal on the abaxial surface compared with the adaxial surface. Interestingly, in addition the abaxial surface of the wing petal possessed tabular stepped cells, with striations particularly noticeable at the borders of each cell, which were located around the petal fold at the base of the flower (Fig. 3). Tabular rugose striate cells were occasionally present over a small proportion of the abaxial surface of the wing petal, towards the base, but the shape and distribution of these cells was not consistent. Standard petals had no unique cell types, with tabular rugose striate cells the predominant cell type on both sides, and tabular rugose smooth cells occasionally present. However, the tabular rugose striate cells at the base of the standard petals, where the corolla-tube is formed, were much more elongate than seen on any other petal (Fig. 2E).

**Table 1:** The distribution of epidermal cell types in *V. faba* flowers. The presence and absence of epidermal cell types previously reported in as a major cell type in legume taxa by Ojeda et al. (2009) (underlined) and additional legume cell-types found in this study on the adaxial and abaxial surface of standard, wing and keel petals are recorded with + or – respectively. ^*^Tabular rugose striate cells were occasionally observed on the abaxial surface of the wing petal in some lines of *V. faba,* but they represented a very small proportion of the cell population and did not have a consistent shape between lines.

Petal type	Tabular cells					Papillose cells		
	Rugose Granular	Rugose Striate	Flat Striate	Rugose Smooth	Stepped Striate	Conical Striate	Knobby Rugose	Lobular Striate
Standard (abaxial)	–	+	–	+	–	–	–	–
Standard (adaxial)	–	+	–	+	–	–	–	–
Wing (abaxial)	–	+^*^	–	+	+	+	–	–
Wing (adaxial)	–	–	–	+	−	+	–	–
Keel (abaxial)	+	+	+	+	–	–	–	–
Keel (adaxial)	+	+	+	+	–	–	–	–

### 3.2 The variation in the distribution of conical cells on the wing and standard petals of *V. faba* lines

**Fig. 4:**
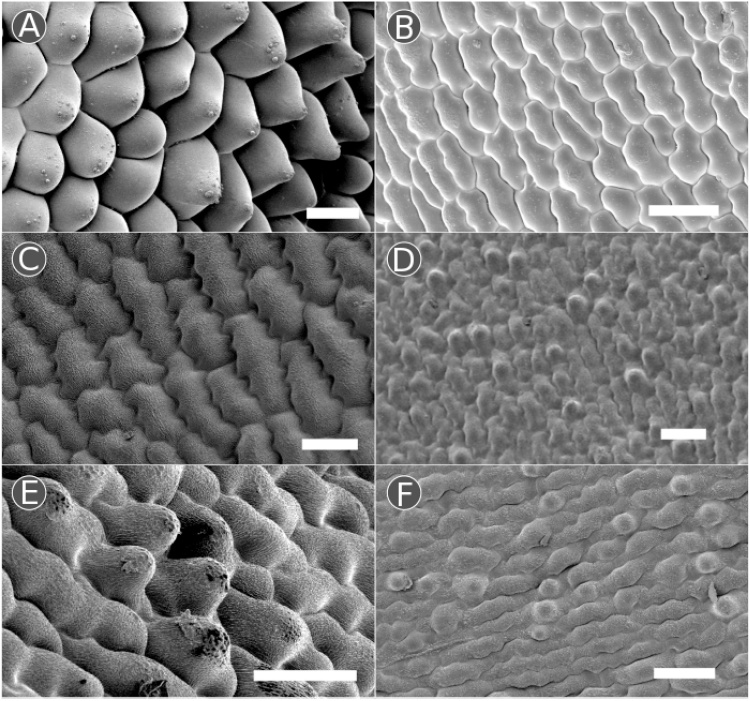
Intraspecific variation in the distribution of conical cells across the wing and standard petals of *V. faba*. Conical cells were most frequently observed across the majority of the adaxial wing petal as seen in line NV574 (A), however line NV639 had no conical cells present on the adaxial surface of the wing petal (B). The abaxial surface of the standard petal was non-conical in epidermal morphology for the majority of lines, as seen in NV754 (C), however lines NV175 (D), NV676 (E), and NV868 (F) had a patchy distribution of conical cells across this petal. Scale bars: (A, C, E) 50 μm; (B, D, F) 100 μm.

As the aim of this study was to identify intraspecific variation in traits which may affect the pollination of *V. faba*, following the detailed investigation of the epidermal cell types present in a flower, a more focused study of an additional 27 randomly selected lines was undertaken. This wider survey centered on the distribution of papillose conical striate cells (from here on termed ‘conical cells’) due to their known potential to affect bee preference (Whitney et al., 2009; Alcorn et al., 2012). Of the 32 lines examined in total (including those described in Section 3.1), the majority (28 lines) exhibited a similar distribution of cell types across the flower (Table 2; Fig. 3). This consisted of a non-conical celled standard petal, on both the adaxial and abaxial surface, and conical cells across the majority of the wing petals, on both the adaxial and abaxial surface. However, interestingly, we detected four lines that had deviations from this pattern (Figs. 3 and 4 ). Conical cells were absent from the adaxial side of the wing petal in line NV639, although present on the abaxial epidermis. Furthermore, in three lines, NV175, NV676 and NV868, patches of conical cells were identified on the abaxial, but not adaxial, surface of the standard petal. Both of these phenotypes were consistent within a genotype when two further plants each of lines NV639 and NV676 were examined, although the extent of the conical cell patches in NV676 varied in size. Curiously, all three of the lines with conical cell production on the standard lack the black-brown wing petal spot present in most *V. faba* genotypes (Table S1). However, the two other ‘non-spotted’ lines surveyed, NV643 and NV644, did not have abnormal distributions of conical cells.

**Table 2:** A summary of the distribution of conical cells across the surface of the petals of *V. faba* lines. “Conical” indicates the presence of conical cells; this was the major cell type on the wing petals unless indicated otherwise. Where the standard petal is indicated as “Conical” this was not necessarily the main cell type, see the main text for further details. Abnormal phenotypes are highlighted with emboldened text.

Line	Standard petal (adaxial)	Standard petal (abaxial)	Wing petal (adaxial)	Wing petal (abaxial)
NV002	Non-conical	Non-conical	Conical	Conical
NV020	Non-conical	Non-conical	Conical	Conical
NV027	Non-conical	Non-conical	Conical	Conical
NV079	Non-conical	Non-conical	Conical	Conical
NV082	Non-conical	Non-conical	Conical	Conical
NV100	Non-conical	Non-conical	Conical	Conical
NV129	Non-conical	Non-conical	Conical	Conical
NV155	Non-conical	Non-conical	Conical	Conical
NV175	Non-conical	**Conical**	Conical	Conical
NV293	Non-conical	Non-conical	Conical	Conical
NV490	Non-conical	Non-conical	Conical	Conical
NV574	Non-conical	Non-conical	Conical	Conical
NV604	Non-conical	Non-conical	Conical	Conical
NV619	Non-conical	Non-conical	Conical	Conical
NV620	Non-conical	Non-conical	Conical	Conical
NV626	Non-conical	Non-conical	Conical	Conical
NV639	Non-conical	Non-conical	Conical	**Non-conical**
NV640	Non-conical	Non-conical	Conical	Conical
NV641	Non-conical	Non-conical	Conical	Conical
NV643	Non-conical	Non-conical	Conical	Conical
NV644	Non-conical	Non-conical	Conical	Conical
NV648	Non-conical	Non-conical	Conical	Conical
NV649	Non-conical	Non-conical	Conical	Conical
NV650	Non-conical	Non-conical	Conical	Conical
NV653	Non-conical	Non-conical	Conical	Conical
NV658	Non-conical	Non-conical	Conical	Conical
NV671	Non-conical	Non-conical	Conical	Conical
NV673	Non-conical	Non-conical	Conical	Conical
NV675	Non-conical	Non-conical	Conical	Conical
NV676	Non-conical	**Conical**	Conical	Conical
NV706	Non-conical	Non-conical	Conical	Conical
NV868	Non-conical	**Conical**	Conical	Conical

During our survey of variation in conical cell production across different genotypes, we also noted more subtle differences in cell shape between different lines (Fig. S1). In some lines, such as NV706, typical conical cells were interspersed with more rounded cells that lacked striations. Furthermore, the ratio between cell height and width appears to vary widely, even within a flower, such as seen on the wing petals of NV673, where the conical cells appear to have roughly a height to width ratio close to one on the adaxial surface but much greater than one on the abaxial surface.

## 4 Discussion

Petal cell epidermal morphology has an important function in mediating the interaction of a flower with pollinators as well as potential antagonists such as nectar robbers (Papiorek et al., 2014). In this paper we provide the first comprehensive study of intraspecific variation in the distribution of petal epidermal cell types, in this case within an important crop species, *V. faba.* During a detailed study of five genotypes, we built on the previous examination of *V. faba* floral epidermal cell morphology by Ojeda et al. (2009). We determined that each petal surface has a unique combination of cell types, presumably because each surface performs a different function and interacts with different flower visitors in different ways.

Previously, intraspecific differences in the petal epidermal morphology have only been reported between two subspecies of *Echium wildpretii* with different functional groups of pollinators (Ojeda et al., 2016). “Negligible” intraspecific petal micro-morphological variation has been identified within the legume species *Lotus japonicus* (Regel) K.Larsen, and *Trifolium repens* L. (3–4 plants; Ojeda et al., 2009), and while multiple plants of six *Lathyrus* L. species were examined by Çildir et al. (2012; 3–6 plants), they do not describe any intraspecific differences between individuals. Here, we report a much higher level of intraspecific variation in the distribution of a functionally important cell type for the interaction of a flower with pollinators; conical cells. Across the 32 genotypes of *V. faba* that we studied, ∼ 13% (4/32) had a substantially different distribution of conical cells. This level of variation is lower than that seen at the intrageneric level: previous studies report an average of ∼ 40% of petal epidermal phenotypes deviating from the most common phenotype within a genus (Kay et al., 1981; Ojeda et al., 2009, 2012, 2016; Çildir et al., 2012; Papiorek et al., 2014; Supplementary file 2). A lower level of variation might reflect the adaptively significant nature of petal epidermal cell shape, with individual species generally presenting a particular phenotype that functions in concert with their particular pollination system. However, the presence of a considerable degree of intraspecific variation means that pollinators will fairly commonly encounter variation, providing opportunities for natural selection to act and for species to diverge.

During our examination of the distribution of conical cells across the standard and wing petals of a large number of genetically distinct lines we found two abnormal phenotypes. Of particular interest is the observation that line NV639 has no conical cells on the adaxial face of the wing petal, compared with an abundance of conical cells localized towards the tip of this surface in all other lines assessed. In addition, three of the non-spotted lines, NV676, NV868 and NV175, have a normal conical cell distribution on the wing petals, but the abaxial side of the standard petal also has a patchy distribution of ectopic conical cells. The differences in phenotype we identified during this study were therefore petal-type specific, and petal surface (ad/abaxial) specific, rather than loss or gain of function across the entire flower. This suggests that the genetic basis of these changes lies within alteration of gene expression patterns rather than loss or gain of protein function. Conical cell development is regulated by members of the Subgroup 9 R2R3 MYB transcription factor family, containing *MIXTA, MIXTA-like, MYB17* and *MYB17-like* genes (Noda et al., 1994; Brockington et al., 2013). In *Thalictrum* loss of conical cells with the transition to wind pollination has been associated with changes in expression of *Mixta-like2* (Di Stilio et al., 2009). Furthermore, Brockington et al. (2013) demonstrated that a *MYB17* gene from *L. japonicus* could induce epidermal cell outgrowth into conical forms when expressed ectopically. However, changes in petal shape and the distribution of conical cells in the bird pollinated section of *Lotus* (*Rhyncholotus* (Monod) D.D.Sokoloff), such that they are lost from the standard petal and located only towards the base of the abaxial wing petal surface, are associated with changes in the timing in expression of *CYC2* (Ojeda et al., 2012, 2017). In *L. japonicus*, *LjCYC2* determines petal-type within a zygomorphic flower, activating the transcription of Subgroup 9 MYB genes to promote conical cell development in the dorsal (standard) petal (Feng et al., 2006). In the flowers we identified with an abnormal phenotype there were no obvious changes in the shape of petals. This suggests that changes in the expression pattern of downstream targets such as the MYB Subgroup 9 genes, rather than genes regulating petal position within the flower, are more likely to be responsible for the range of phenotypes seen.

The distribution of conical cells within a flower has the potential to affect the ease with which pollinators can manipulate flowers by altering how easily they can grip on to the surface of the flower. In *V. faba*, pollinators tend to contact the abaxial surface of the wing petal and occasionally the adaxial surface of the standard petal when foraging legitimately on the flower (Stoddard, 1991). Therefore the two abnormal phenotypes identified in this study are unlikely to have an effect on the ease of manipulation of the flower by pollinators. On the other hand, the extended distribution of conical cells on the abaxial surface of the standard may make it easier for robbers to forage on the flowers (Papiorek et al., 2014) and therefore have a negative effect on the pollination of the flower as a whole. The majority of the Vicoid clade of legumes, in which *V. faba* is contained, have a similar distribution of cell types across the flower, with conical cells only rarely present on the standard and more frequently found on wing petals alone (Ojeda et al., 2009). Our study therefore suggests that selective breeding within this crop has not resulted in a suboptimal conical cell distribution for the pollination of bean flowers in the large majority of genotypes.

Consistent with a previous investigation of *V. faba* flowers, conical cells were restricted to the wing petals (with the exception of our three genotypes with abnormal phenotypes), where they were the major cell type (Ojeda et al., 2009). Our finer detailed investigations identified cell types that were distributed over a small area of the petals which have previously not been reported in *V. faba,* for example tabular rugose granular cells on the keel petals. Interestingly, a previously undescribed cell type, termed here as tabular stepped striate cells, was identified (Fig. 3). Although these cells were flat in nature, it was thought that their structure into overlapping rows of cells to form a stepped structure merited a discrete category of cell type. These tabular stepped striate cells were found specifically around the folds of the wing petal on the abaxial surface, suggesting that they may have some function in the tripping (flower opening) mechanism of *V. faba*. Our study also indicates there may be more subtle differences in the shape of conical cells between genotypes. These may have an effect on how easily bees can interact with the petal surface by changing how well the tarsal claws of the bee interlock with the conical cells. Our study was not designed to examine these differences in cell shape, however it would be interesting to investigate if these differences are consistent in future studies.

## 5 Conclusions

The results of this study represent the first comprehensive assessment of the level of variation in petal epidermal morphology within a species. From an agronomic perspective, it suggests that for the large part the flowers of the field bean are optimized for pollination by bees; conical cells are present on the abaxial surface of the wing petal, the most important petal surface for a bee manipulating a flower, providing grip in all of the genotypes that we analysed. We found a surprising level of variation in the distribution of conical cells across the flower between genotypes, with ∼13% of flowers showing a substantially different distribution of conical petal epidermal cells. Indeed, this is the first time that intraspecific variation in petal epidermal cell morphology has been reported in Fabaceae. Interestingly, none of these changes involved the loss or gain of conical cells across the flower. This suggests that alterations in distributions of conical cells are more common than flower-wide changes in distribution resulting from loss of gene function, highlighting the importance of molecular evolution of regulatory regions of the genome in the generation of novel floral morphologies.

## Data accessibility

All data generated or analysed during this study are included in this published article (and its Supplementary Information files).

## Author contributions

Data were collected by E.J.B. Both E.J.B and B.J.G. conceived the study and drafted the manuscript. Both authors gave final approval for publication.

## Competing interests

The authors declare no competing financial interests.
