# Supplementary material for: Intraspecific variation in the petal epidermal cell morphology of Vicia faba L. (Fabaceae)
Source: Flora. 2018 Jul;244-245:29–36. doi: 10.1016/j.flora.2018.06.005 (PMC6039855; doi:10.1016/j.flora.2018.06.005)
Supplement: Supplementary file 1 [file mmc1.doc]

**Supplementary File 2: Intrageneric variation in the epidermal morphology of flowers**

| Data based on genera where more than 4 species reported | | | |  |  |
| --- | --- | --- | --- | --- | --- |
| Reference | Genera | No. spp. | Spp. that deviate | % deviation | Notes |
| Cildir 2012 | Lathyrus | 6 | 3 | 0.5 |  |
| Ojeda 2009 | Cassia | 6 | 1 | 0.166666667 |  |
|  | Bauhinia | 6 | 3 | 0.5 |  |
|  | Senna | 12 | 3 | 0.25 |  |
|  | Genista | 4 | 3 | 0.75 | ie. All diff |
|  | Dalea | 4 | 1 | 0.25 |  |
|  | Dalbergia | 6 | 2 | 0.333333333 |  |
|  | Erythina | 5 | 3 | 0.6 |  |
|  | Lotus | 7 | 1 | 0.142857143 |  |
|  | Lathyrus | 7 | 3 | 0.428571429 |  |
|  | Trifolium | 5 | 1 | 0.2 |  |
|  | **Vicia** | **5** | **3** | **0.6** |  |
|  | Wysteria | 4 | 2 | 0.5 |  |
| Papiorek 2014 | Sinningia | 5 | 3 | 0.6 | threshold of 0.1 diff |
| Kay 1981 | Campanula | 4 | 2 | 0.5 |  |
|  | Potentilla | 4 | 2 | 0.5 |  |
|  | Galium | 5 | 2 | 0.4 |  |
|  | Saxifraga | 8 | 3 | 0.375 | 2 cultivars of unknown species included as different species |
|  | Viola | 4 | 1 | 0.25 |  |
| Ojeda 2012 | Lotus | 49 | 30 | 0.612244898 |  |
| Ojeda 2016 | Lotus | 9 | 4 | 0.444444444 |  |
|  | Thermopsis | 5 | 4 | 0.8 |  |
|  | Scrophularia | 13 | 9 | 0.692307692 |  |
|  | Digitalis | 4 | 1 | 0.25 |  |
|  | Echium | 8 | 4 | 0.5 | 2 subspecies of the same species (pollinated by diff pollinators) included as seprate species |
|  | Laratera | 6 | 0 | 0 |  |
|  | Teucrium | 12 | 7 | 0.583333333 |  |
|  |  |  |  |  |  |
|  |  |  | Average | 0.434398479 |  |
