# Supplementary material for: Intraspecific variation in the petal epidermal cell morphology of Vicia faba L. (Fabaceae)
Source: Flora. 2018 Jul;244-245:29–36. doi: 10.1016/j.flora.2018.06.005 (PMC6039855; doi:10.1016/j.flora.2018.06.005)

Intraspecific variation in the petal epidermal cell morphology of *Vicia faba* (Fabaceae)

Emily J Bailes^1,2,3^, Beverley J Glover^2^

^1^ School of Biological Sciences, Royal Holloway University of London, Egham, Surrey, TW20 0EX

^2^Department of Plant Sciences, University of Cambridge, Downing Street, Cambridge, CB2 3EA

^3^National Institute of Agricultural Botany, Huntington Road, Cambridge, CB3 0LE

*Corresponding author: emilyjbailes@gmail.com; The Bourne Laboratory, School of Biological Sciences, Royal Holloway, University of London, Egham, Surrey, TW20 0EX

**Table S1 – Details of seed source and of herbarium specimens for the lines used in this study.** Information supplied by the National Institute of Agricultural Botany.

| **Line** | **Seed source** | **Donor ref and other names** | **Origin** | **Colour** | **Collector and collection number** | **Herbarium barcode (accession number)** |
| --- | --- | --- | --- | --- | --- | --- |
| NV002 | ICARDA | ig11197 | landrace | white/black wing petal spots | Dorling 1 | CGE33556 |
| NV020 | ICARDA | ig11290 | landrace | white/black wing petal spots | Dorling 2 | CGE33557 |
| NV027 | ICARDA | ig11312 | landrace | white/black wing petal spots | Dorling 3 | CGE33558 |
| NV079 | ICARDA | ig11687 | landrace | white/black wing petal spots | Dorling 4 | CGE33559 |
| NV082 | ICARDA | ig11695 | landrace | white/black wing petal spots | Dorling 5 | CGE33560 |
| NV100 | ICARDA | ig11749 | landrace | white/black wing petal spots | Dorling 6 | CGE33561 |
| NV129 | ICARDA | ig12137 | landrace | white/black wing petal spots | Dorling 7 | CGE33562 |
| NV155 | ICARDA | ig12684 | landrace | white/black wing petal spots | Dorling 8 | CGE33563 |
| NV175 | ICARDA | ig13004 | landrace | white, no petal spots | Dorling 9 | CGE33564 |
| NV293 | ICARDA | ig70726 | landrace | white/black wing petal spots | Dorling 10 | CGE33565 |
| NV490 | ICARDA | ig124213 | landrace | white/black wing petal spots | Dorling 11 | CGE33566 |
| NV574 | ICARDA | ig130638 | landrace | white/black wing petal spots | Dorling 12 | CGE33567 |
| NV604 | JIC | V185, Borington Bulk | landrace | white/black wing petal spots | Dorling 13 | CGE33568 |
| NV619 | NICK-ADV | NA12,  LAN08935 | landrace | white/black wing petal spots | Dorling 14 | CGE33569 |
| NV620 | CSIC | CSIC,  Vf172 | landrace | white/black wing petal spots | Dorling 15 | CGE33570 |
| NV626 | CBP-T | KWS6,  NPZ 7-7301 | landrace | white/black wing petal spots | Dorling 16 | CGE33571 |
| NV639 | GOTTINGEN | 70176/70175, Hedin | commercial variety | white/black wing petal spots | Dorling 17 | CGE33572 |
| NV640 | NIAB | Maris Bead | commercial variety | white/black wing petal spots/purple venation | Dorling 18 | CGE33573 |
| NV641 | NIAB | Fuego | commercial variety | white/black wing petal spots | Dorling 19 | CGE33574 |
| NV643 | POL | Albus | commercial variety | white, no petal spots | Dorling 20 | CGE33575 |
| NV644 | POL | Kasztelan | commercial variety | white, no petal spots | Dorling 21 | CGE33576 |
| NV648 | ICARDA | ig101769, BPL10 | landrace | white/black wing petal spots | Dorling 22 | CGE33577 |
| NV649 | ICARDA | ig101770, BPL11 | landrace | white/black wing petal spots | Dorling 23 | CGE33578 |
| NV650 | ICARDA | ig101771, BPL12 | landrace | white/black wing petal spots | Dorling 24 | CGE33579 |
| NV653 | ICARDA | ig101786, BPL27 | landrace | white/black wing petal spots | Dorling 25 | CGE33580 |
| NV658 | GOTTINGEN | CGN07715 cf-3 (60354-9), closed flower mutant | landrace | cream/black petal spots | Dorling 26 | CGE33581 |
| NV671 | NIAB | Atlas | commercial variety | white/black wing petal spots | Dorling 27 | CGE33582 |
| NV673 | NIAB | Fury | commercial variety | white/black wing petal spots | Dorling 28 | CGE33583 |
| NV675 | NIAB | Pyramid | commercial variety | white/black wing petal spots | Dorling 29 | CGE33584 |
| NV676 | NIAB | Tattoo | commercial variety | white, no petal spots | Dorling 30 | CGE33585 |
| NV706 | Thompson & Morgan | Broad Bean Crimson Flowered | horticultural variety | crimson/magenta, brown-black petal spots | Dorling 31 | CGE33586 |
| NV868 | Olaf Sass, NPZ | Taifun | commercial variety | white, no petal spots | Dorling 32 | CGE33587 |

**Figure S1** **- The epidermal morphology of *V. faba* flowers**

A representative image of the major cell type found on the adaxial and abaxial surface of the wing and standard petals in each of the 32 lines assessed. Line numbers are indicated below each row of images and scales are given below each image.


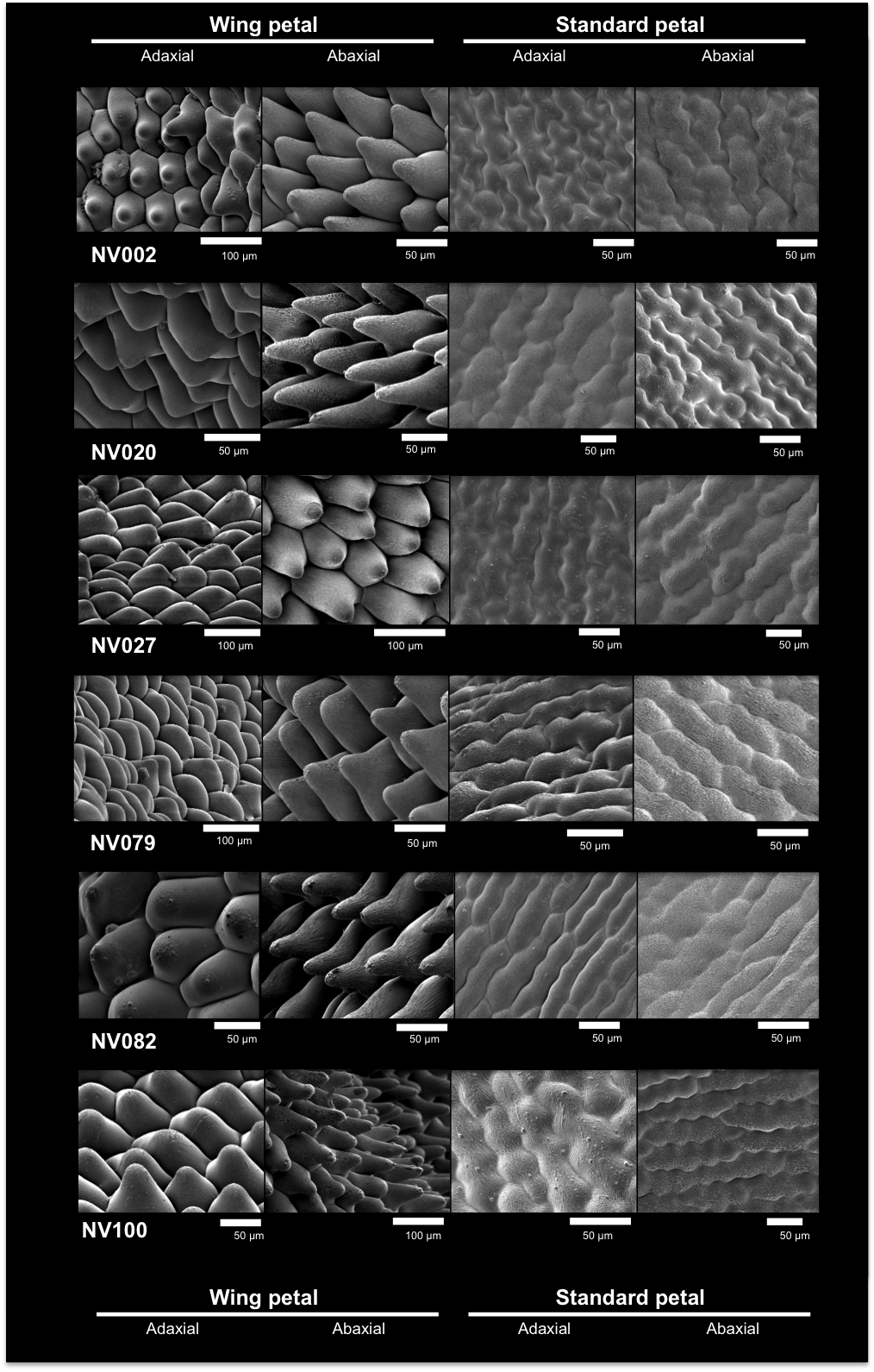


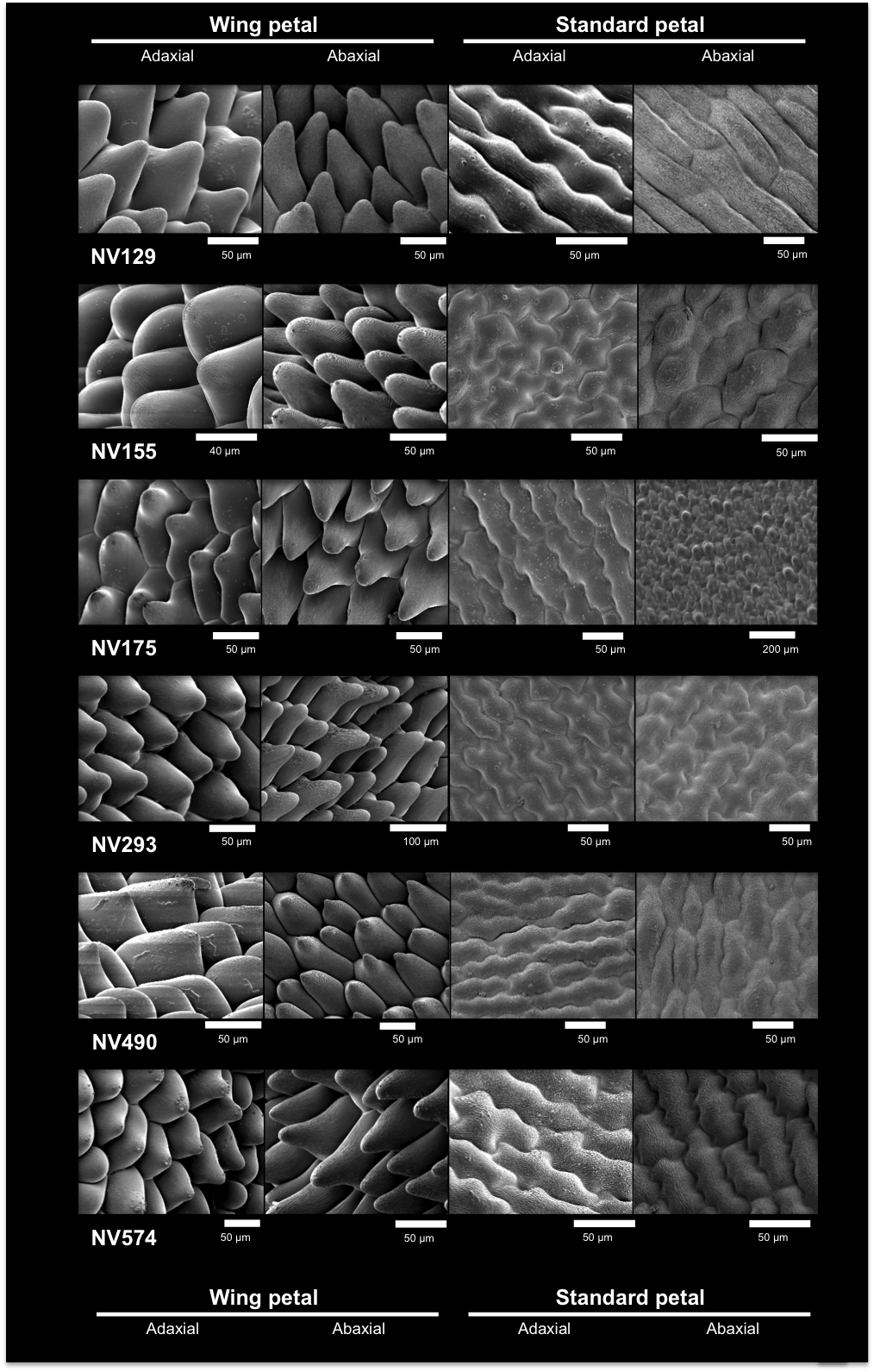


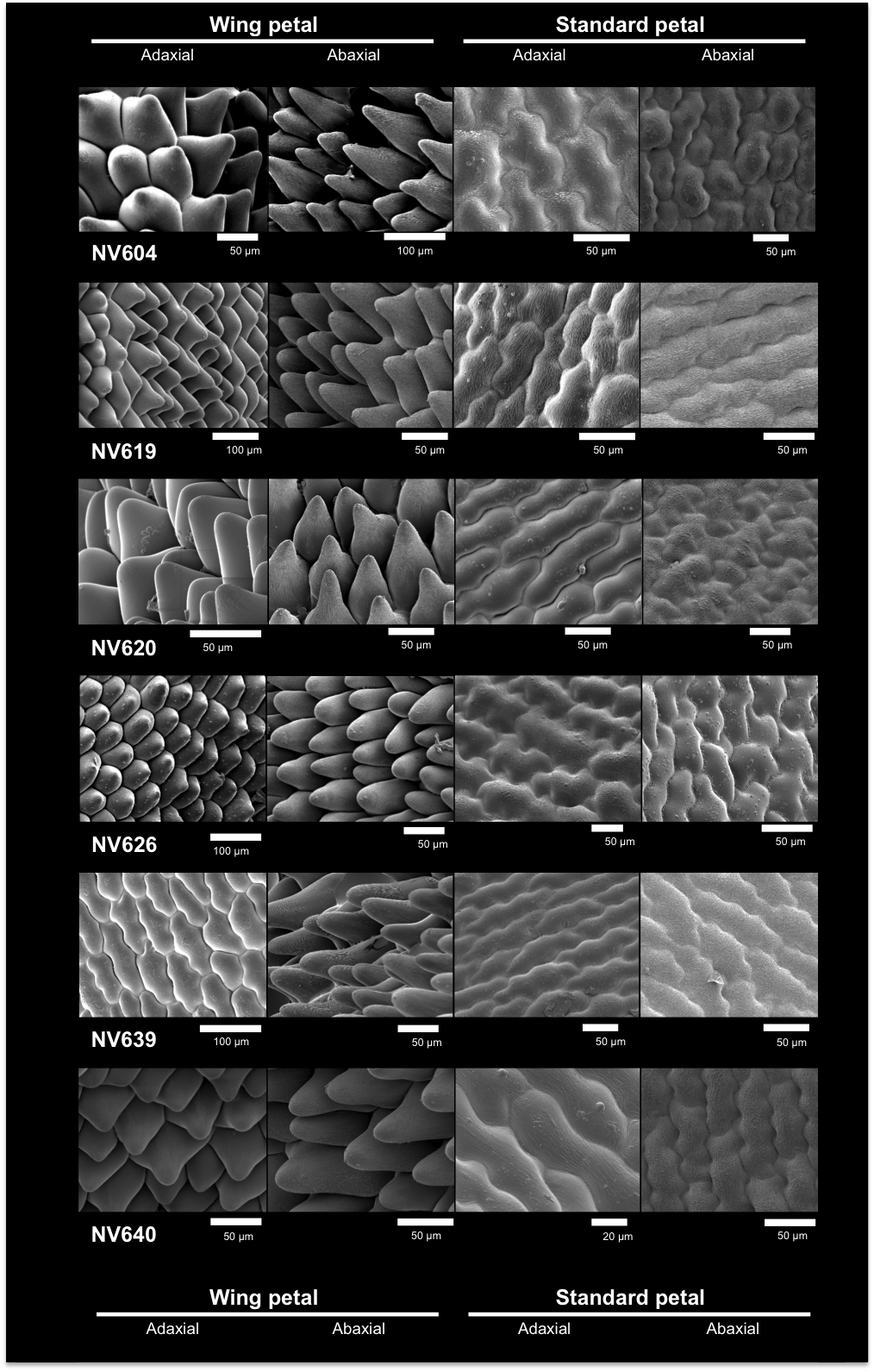


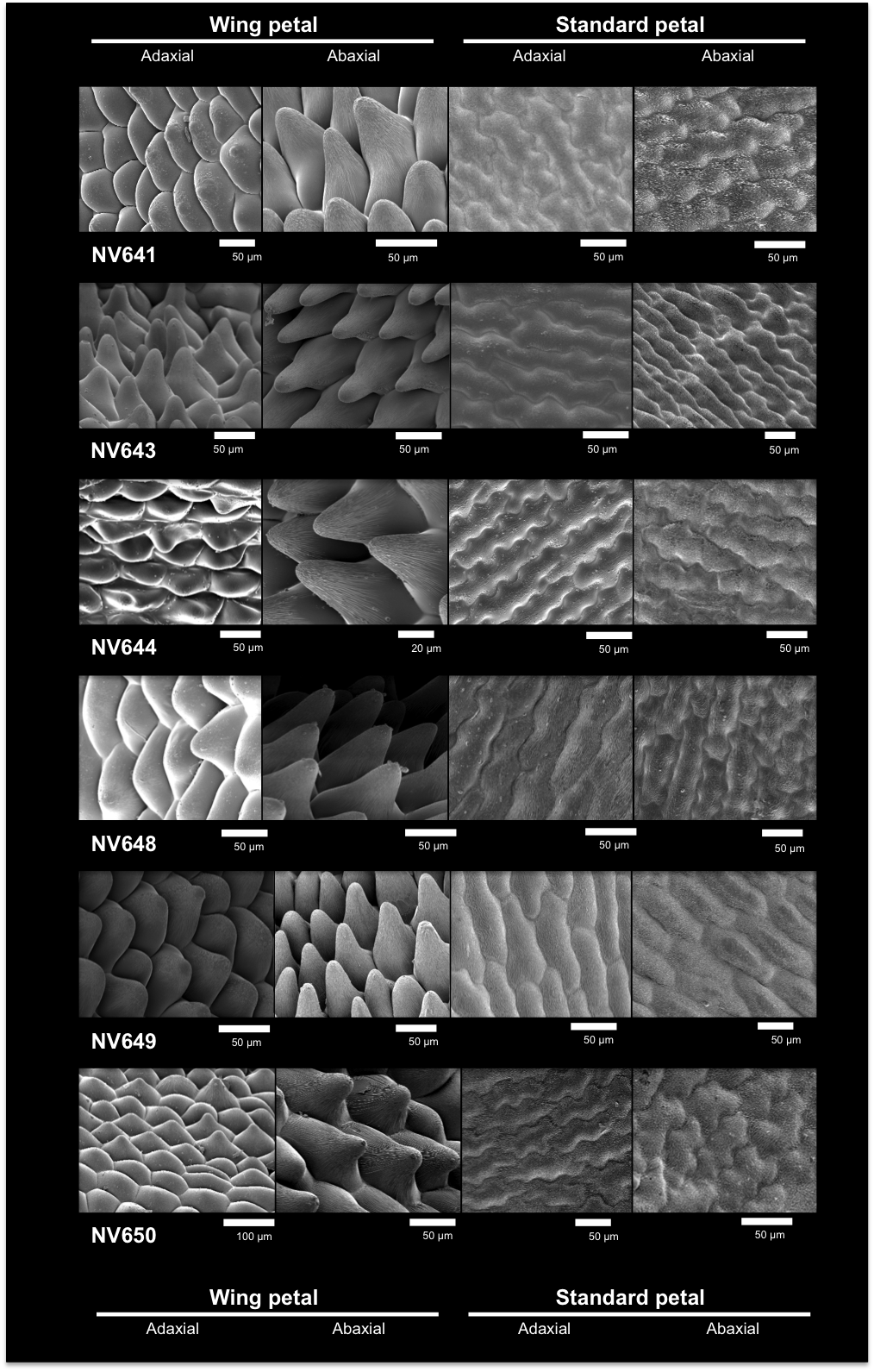


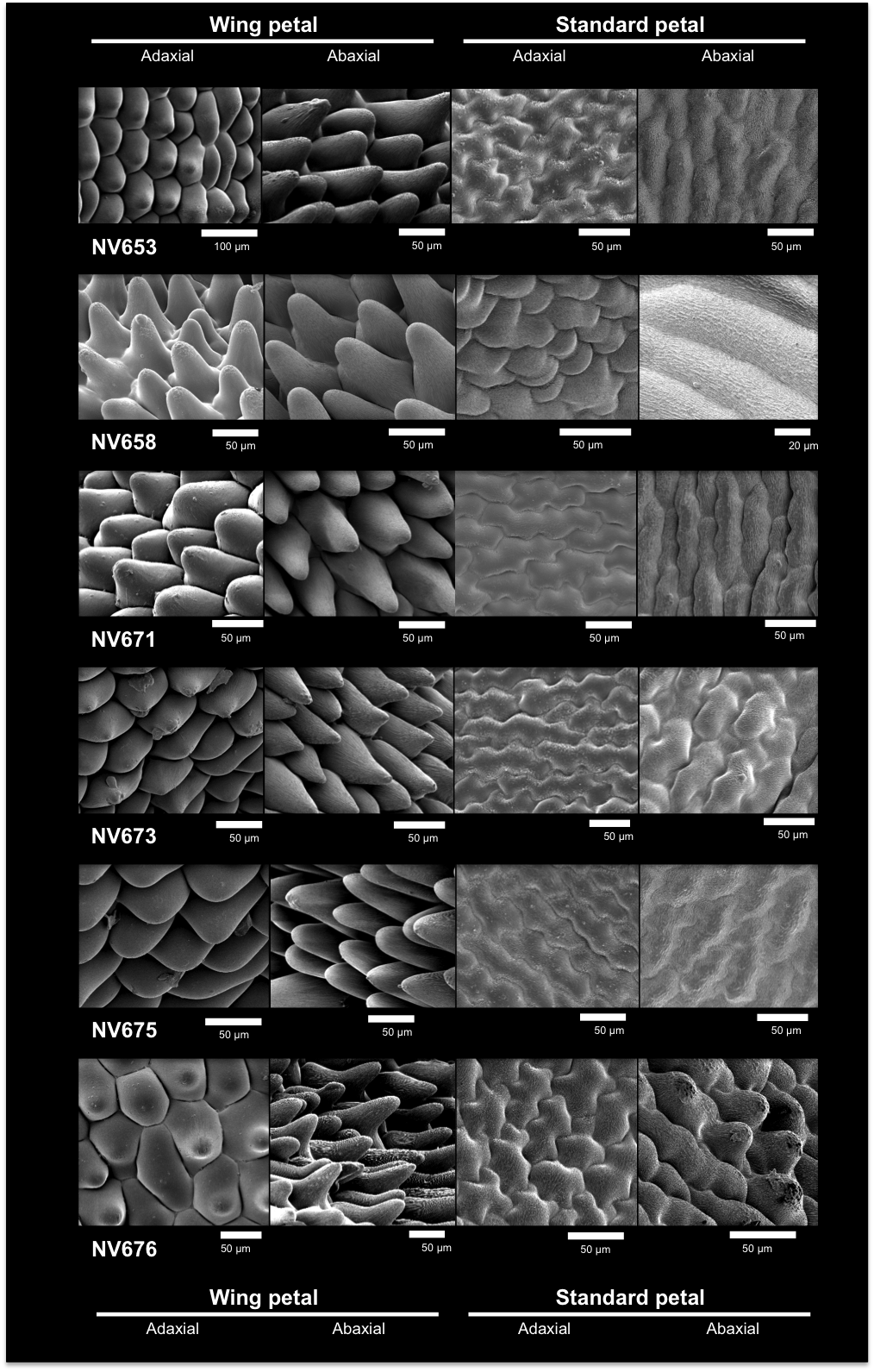


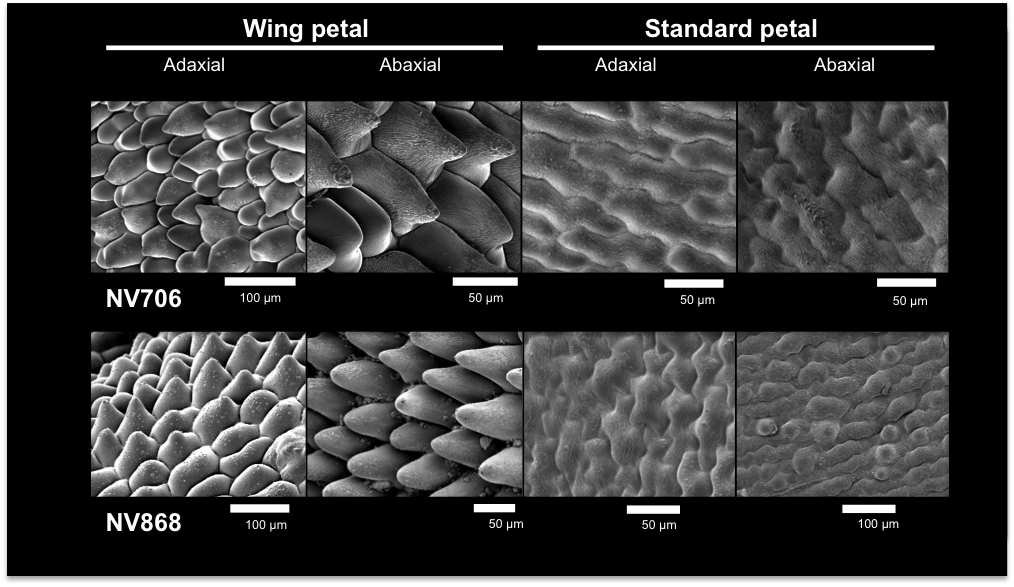

Supplement: Supplementary file 2 [file mmc2.docx]
